# Determinants of Rural Household Food Security Status in North Shewa Zone, Amhara Region, Ethiopia

**DOI:** 10.1155/2022/9561063

**Published:** 2022-04-12

**Authors:** Abebaw Hailu Fikire, Mesele Belay Zegeye

**Affiliations:** Department of Economics, College of Business and Economics, Debre Berhan University, Debre Berhan, Ethiopia

## Abstract

Food insecurity is one of the most serious problems in developing countries, especially in Ethiopia. Therefore, it is important to understand the barriers to improving the state of food security in the country. Thus, this study aims to investigate the determinants of food security of rural households in the North Shewa zone in the Amhara region, Ethiopia. A sample of 796 farm households was considered. This paper used the calorie intake method per day to measure household food security status and a logit model to investigate the determinants of food security. The results show that family size, age of the household head, educational level of the head, off-farm activities, monthly income of the household, and distance from the market are the major determinants of rural household food security in the North Shewa Zone. The findings suggest that expanding the access to education in farm households, expanding the access to off-farm activities to increase household income, and expanding market access to farm households are important to improve rural food security status in the study area.

## 1. Introduction

Nowadays, food security issues become one of the critical concerns and top priority areas for both developed and developing countries [1]. This shows equal importance for both developed and developing countries. According to the Food and Agriculture Organization of the United Nations (FAO) report, 815 million people worldwide are malnourished, a trend that is even worse than before. The majority of malnourished and food insecure people are from developing countries. Therefore, a central policy issue for food-insecure regions of the world, concentrated in Asia and Africa, is how best to respond to the reality of food insecurity [2]. Ethiopia is one of the sub-Saharan African countries, in which the problem of food insecurity has become one of defining features of the country and is repeatedly referred to as food insecure country [1]. This is because of the highest population growth and land degradation, crop and market failures accompanied with droughts and other environment factors, political factors along with low access to assets, the incidence of poverty, and deprivation [3]. In addition, even if agriculture is the mainstay of the economy, its contribution is declining over time, and its productivity is very low as well due to its heavy reliance on rain-fed farming, traditional farming, adverse climatic conditions, and minimal applications of farm technologies. The lower food production and productivity limits the national food availability and consumption requirements, which result in food insecurity problem in the country [4]. As a result, the daily calorie consumption in Ethiopia is below the recommended daily allowance of 2100 kcal/person/day [3]. Thus, food insecurity remains highly prevalent in the country, and over the past two decades, it has increasingly been recognized as a serious public health problem. Furthermore, nowadays, about 8.5 million people are highly food insecure due to the impact of desert locusts, crop failure and displacement, ongoing impacts of drought, and high food prices in the country [5].

Amhara national regional state of is one of the severely affected regions of Ethiopia. The region is the second populous region and agriculture is the main economic activity. The intensive use of land in the region has led to the recurrent occurrence of drought, and this has resulted in 14.8 percent of the rural households being chronically food deficient UNICEF [6]. In the region, 25% of the households were mainly in neighboring rural areas, with one or more members looking for a job during the dry season. One in three immigrants had a hard time finding a job, but half did not bring food or income to their families [7]. The households could not cover their minimum daily calorie from income generated from agriculture as well as from other activities where their participation was found to be low and livestock possession as it has problems of both quantity and quality. Lack of participation in agriculture, farmland scarcity, poverty, recurrent drought and climate change, rainfall shortage and land degradation, population pressure, livestock ownership, distance from input market, nonfarm income, large family size, low annual yields, small farm size, dependence on food aid, poor welfare, and land tenure instability were identified as factors of food insecurity in the region [8–11] [12].

Similarly, North Shewa zone is one of the zones of Amhara region, and the zone has experienced acute food insecurity problem and most of the population of the zone has received only limited assistance from humanitarian organizations. Besides, the per capita growth of production of major food items has not been sufficient to satisfy the demand of an increasing population in the zone. There is a growing concern that the situation may worsen [9, 13, 14] [12]. To this end, to gain more insight on the factors that determine the food security, this study looks at the determinants of household food security in rural North Shewa zone, Amhara region, Ethiopia.

Various studies have been conducted on investigating the determinants of food insecurity at regional and national level [10, 13, 15–19]; Burley, 2013; [9]; and [12]). A lot of similar studies have been done in Ethiopia at both the national and household level. The contribution of this study to the existing literature is twofold: first, studies regarding North Shewa zone are scanty. For example, Hilemelekot et al. [12] and Cheber [9] studied the issue of food security status in the face of climate variability. However, these studies are not enough to show the determinants of food insecurity. Hence, this study investigates the determinants of household food security status in North Shewa Zone. Second, most previous studies confirmed that different factors affect the food security status of smallholders in different parts of the country [9, 13, 15–18] [12]. This advises that location-specific studies that account for unobservable differences in sociocultural, institutional, and economic features among different parts of the country will help government practitioners to make informed decisions. In this regard, using unique primary data from the zone, this study specifically analyzes the determinants of food security status in North Shewa Zone by adopting a food calorie intake approach. Therefore, this study is motivated to analyze the determinants of food security in the rural households of North Shewa Zone, Amhara region, Ethiopia.

## 2. Literature Review

The concept of food security began 30 years ago at the First World Food Conference in the mid-1970s, and its scope and definition were narrow. Initially, the concept focused on national and international interest and was defined in terms of food supply, paying particular attention to stable food prices and food availability [20]. Food security exists when everyone always has physical, social, and economic access to sufficient, safe, and nutritious food that meets their dietary needs and dietary preferences and dietary needs for an active and healthy life. The definition of food security as previously mentioned consists of four dimensions, namely, access to food, food availability, use, and sustainability [21]. Consequently, still more than 8.5 million people are undernourished, and almost all of them belong to the developing countries [2]. Sub-Saharan Africa (SSA) has the highest number of people living in extreme poverty, comprising 413.3 million people in 2015. Although there have been efforts to achieve food security at the household level in Ethiopia, nearly 25 percent of the population still lives below the nationally defined poverty line [22]. Bewket [23] stated that once every three or four years is a drought year in Ethiopia. Environmental degradation is also a critical factor, which exacerbates soil loss, deforestation, and pest incidence, all of which affect food security. In addition, rapid population growth, poverty, rural-urban migration, and conflict can contribute to food insecurity.

Food insecurity became a key problem and development challenge for Ethiopia in the early 1970s and became widespread in the decades that followed. More importantly, since the 1980s, severe droughts and massive famines have triggered the need for food security and food aid initiatives. Conversely, the concept has become more complex as the level of analysis moves from global and national to household and individual levels [24]. The severity of food insecurity problem in Ethiopia varies from region to region depending up on natural resources availability. Drought is the only significant cause of chronic food insecurity in Ethiopia. The most affected regions by drought and food insecurity are mainly Tigray, Amhara, Afar, Somalia, and some parts of Oromia regions [25].

The problem of food insecurity in different parts of the world in general and particularly in Ethiopia is caused by different factors; empirical evidences showed that food insecurity is caused by low per capita income; low and unequal income distributions affect food, particularly low volatile growth rates in agriculture, unemployment and underemployment, small or declining farm size, inequality of domestic distribution, low land use, social discrimination, population growth, market access, food taboos: certain restrictions on food consumption, poverty and climate insecurity due to cultural and social norms, climate change, deforestation, landslides, reduced soil fertility, political instability, poverty, marginalization, ethnic and low-caste groups, and high maternal and infant mortality ([21]; Beyene and Muche, 2010; [10, 13, 17–19]; Burley, 2013; [9]; and [12]). Furthermore, the problem of food insecurity is not only caused by insufficient supply of food, but also due to the lack of purchasing power and access at national and household levels. Abafita and Kim [15]; Abera [26]; Astemir [27] and Habtewold [28] revealed that household head age and education level, rainfall shock and household size, farmland size, land quality and credit, farm income, fertilizer use and access to bull ownership, education level, land ownership, technology adoption, economic activity, off-farm participation, soil conservation practices and per capita consumption expenditures, access to credit and remittances, and distance from markets were identified as major determinants of food security in different parts of Ethiopia. In general, for long decades, food insecurity continues to be one of the major problems challenging the country Ethiopia. Therefore, reducing the determinate of food security is an important strategy for rural households to achieving food self-sufficiency and poverty reduction among rural households.

## 3. Methodology

### 3.1. Study Area Profile

**Figure 1:**
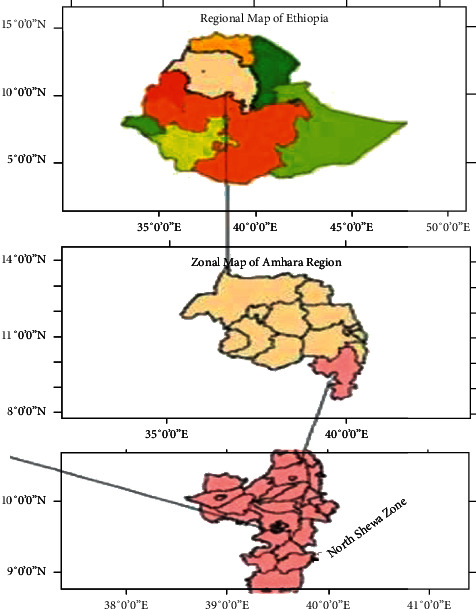
Geographical Location of North Shewa zone; source: Fikire [31].

The study area, North Shewa Zone, is one of the zones of Amhara National Regional State. The zone is bordered by the Oromia region to the south and west, South Wollo to the north, Oromia region to the northeast, and the Afar region to the east. Geographically, the zone is located between 8° 38′ and 10° 42′ north latitude and 38° 40′ and 40° 03′ east longitude and consists of 22 rural areas. According to CSA [29], North Shewa has a population of 2.16 million, of which 50.5% are male and 49.5% are female. The total area of the land plot is 15936.13 square kilometers. Agriculture is the mainstay economic activity, in which nearly 90 percent of the population makes their livelihood from agriculture. The most common agricultural activities practiced in the region are crop production, plantation, animal husbandry, forestry and logging, and fishing. Despite its importance, agriculture in the zone is challenged by factors such as moisture stress, soil erosion, shortage of arable land, draught power shortages, high incidences of pests and diseases, annihilating human and livestock diseases, the untimely supply of meager agricultural inputs, and poor weeds management. This, in turn, has aggravated the food insecurity problem in the area [30]. The geographical location of the zone is presented in Figure 1.

### 3.2. Data Description

This study uses data collected from primary and secondary sources. This study is based on household-level data collected from farm households in North Shewa Zone through a well-structured questionnaire. The questionnaire was designed to collect data on the demographic, economic, social, and institutional characteristics of farm households. More importantly, a section of the survey deals with different consumption bundles, which is later, used to calculate the food calorie intakes of the farm households. Secondary information was collected from documented and published sources such as books, journal articles, conference proceedings, and reports from the Northern Shewa Agricultural office.

### 3.3. Sampling Method and Size

For this study, samples were collected using a multistage sampling method. First, the four districts of Minjar-Shenkora, Angolela Tera, Moretna-Jiru, and Menz-Gera were deliberately chosen due to their high agricultural potential and topographical similarity. Second, 30 Kebeles were randomly selected from the four districts. According to the administration of Northern Shewa District (2019), there are a total of 117,149 households in the selected district. With this in mind, Vogel [32] and Malhotra [33] suggested that between 35000 and 150,000 for a largely homogeneous population, researchers could select a representative sample of up to 800 respondents. Third, in this study, 800 households were selected from the four districts. Finally, simple random sampling was used to select each respondent from each selected Kebele. Due to missing information, four observations were dropped. Thus, the final sample size of the study is determined to be 796 farm households.

### 3.4. Methods of Data Analysis

This study used both descriptive and econometric methods to analyze the data. Descriptive analysis such as mean, standard deviation, minimum, and maximum is used to better understand the demographic, socioeconomic, and institutional characteristics of farm households, and an econometric method through binary logistic regression is used to estimate the determinants of food security in the study area.

### 3.5. Food Security Measures

In this study, the food security status of households is measured by the calorie intake method. Based on the calories intake method, the food security line is defined by selecting a “basket” of food items usually consumed by the households. The amount of the basket is decided in such a way that the given bundle meets the predetermined level of minimum caloric requirement by the Ethiopian government such that 2200 kcal [34]. This “basket” is the estimated the amount of total food consumed over the last seven days of the survey period. To calculate how many calories a household must consume to meet food security requirements, the amount of grain consumed was converted into grams and the calorie content was estimated using the nutritional scheme of foods commonly consumed in North Shewa zone. This method yields a representative food security line. Then, using the 2200 kcal threshold line, we classified the sampled households as food secure and food insecure using the minimum acceptable weighted average food requirement per adult equivalent (AE) per day. The adult equivalent conversion factor takes into account the age and sex of each household member [35]. This means that households with a daily caloric intake of 2,200 kcal or more per adult are considered as food secured, and households with less than 2,200 kcal are considered as food insecure.

### 3.6. Model Specification

In this study, the dependent variable (food security status) is dichotomous, which takes a value of 1 if a household is food secure and the value of 0 if the household is food insecure. Hence, we have two appropriate models at disposal, namely, logit and probit. In practice the logit model is simpler in estimation than the probit model and the logit model also provides more stable results than the probit estimation [36]. Therefore, we used the logit model to estimate the results of the study. Here, we are interested in estimating the probability that a household is food insecure, given the explanatory variables. Following Gujarati [36], the binary logistic model is expressed as

(1) $$\begin{array}{c} L_{I}=\ln\left(\frac{P\left(Y_{i}=1/X_{i}\right)}{1-P\left(Y_{i}=1/X_{i}\right)}\right)=Z_{i}=\alpha +\beta_{i}X_{i}+\varepsilon_{i}, \end{array}$$

where *Z*_*i*_ represents the dependent variable (household food security status). *α* is the intersection of the models. *ß*_*i*_ represents the unknown coefficient to estimate. *X*_*i*_ is the vector of independent variables that can influence the dependent variable, and *ε*_*i*_ is the perturbation condition of the model. It should be noted that the estimated coefficients do not directly indicate the effect of change in the corresponding explanatory variables. Thus, the study estimates marginal effects to indicate the effect of change in explanatory variables on the probability (P) of the outcome occurring.

#### 3.6.1. Description, Measurement, and Hypothesis of the Study

Table 1 presents the description and measurement of variables used in the regression and hypothesis of the study.

## 4. Result and Discussion

### 4.1. Introduction

This section presents the descriptive and regression analysis results of the study. Descriptive analysis used tools such as mean, percentage, standard deviation, and frequency distribution. Logit model was used to determine the determinants of food security in the Northern Shewa region.

### 4.2. Descriptive Analysis

#### 4.2.1. Demographic Characteristics

Table 2 shows that the average family size of the sampled households is five per family. The average age of the sampled household heads was 43 years and the standard deviation is 11.026. The maximum age observed was 80 years and the minimum age was 18 years. The average total arable land for the sampled households is 1.581 hectares, with a standard deviation of 1.101 hectares. The minimum observation area of arable land was 0.25 ha and the maximum was 10 ha. The average distance from home to the nearest market is 9.892 km with a standard deviation of 9.148 minutes. 0.01. And the maximum distance to the market is 70 km. The average livestock asset in terms of tropical livestock is 6.263 with a standard deviation of 4.32, with a minimum of 0 and a maximum of 44.6. Finally, the average monthly income of the household is 3914.5 birr.

Table 3 shows that 90.08% are male-headed households, while only 9.92% are female headed households. From the sampled households, about 46.61% have credit access, while 53.39% households do not have credit access. The information presented in Table 3 also shows that about 38.07% of the respondents were engaged in off-farm activity, while the remaining 61.93% were not. Regarding to saving, 77.26% do not make saving a habit, and 22.74% save part of their income in formal and informal financial institutions. Finally, about 38.82% respondents are no attended formal education, of which 48.37% graduate from primary school, 90 (11.31%) attend secondary school, and 1.51% is above secondary school.

#### 4.2.2. Food Security Status in North Shewa

Table 4 shows that based on the calculated food security threshold line 2200 kcal, a total of 482 (60.55%) of sample households were food insecure and a total of 314 (39.45%) of sample households were food secure in the study area.

### 4.3. Econometrics Analysis

#### 4.3.1. Determinants of Rural Household Food Security Status

Table 5 presents the main findings of this study (such that determinants of food security status). The estimated results revealed that the overall fitness of the model is significant at 1% (Prob >chi^2^ = 0.0001). The table presents the marginal effects of the determinants of the food security situation of rural households in the study area. The results show that keeping other things unchanged, the variable educational level of the household head has a positive and significant effect on food security status, indicating that householders that are more educated can make more informed decisions about consumption and the importance of healthy eating. In addition, these households are better able to access and use social programs to improve their nutrition. The marginal effect indicates that as the number of years of schooling increases, the probability of food security increases by 2.1% relative to the counterpart. This result is in line with [1, 11].

The result shows that keeping other things remains unchanged, the variable family size has negative and significant effect on rural household food security status, and this implies that increasing family size, having less active household members, increases the number of dependent members in the family, increases consumer spending, and reduces the ability to provide enough food for a household. Furthermore, increase in the household size puts more burdens on earning members and may affect their food security status due to availability of limited resources. The marginal effect shows that an increase in the household size by one member decreases the probability of a household being food secure by 13.3%, compared with other subjects, all other things being equal. This result is consistent with [13].

The empirical analysis shows that keeping other things unchanged, the variable distance to market has a negative significant effect on food security status of the households. This indicates that households are both consumers and producers as they have to travel long distances to buy agricultural inputs, consumer goods, and sell their agricultural products, costing farmers money, and they would be food secure by consuming their own product. The marginal effect indicates that a household is located one kilometer away from the market; the probability of food security will decrease by 4.6% compared to households. This result is consistent with [1, 13]. The empirical analysis shows that the variable off-farm activities participation has a positive and significant effect on food security status of the households; this implies that participating off farm economic activities can enable farm households to have diverse sources of income sources and they can improve their wealth situation and help them to improve food security status. This result is consistent with [11].

The results show that keeping other things unchanged, the variable age of household heads also has a positive effect on food security status; this is possible because farmers get more and more experience in their farming operation, climatic knowledge of their area, accumulate wealth, and use better planning than the younger ones. Hence, they have better chance not to become food insecure. Regarding the marginal effect, keeping other factors unchanged, one year increase in the age of the household head will result in an increase the probability of being food secure by about 0.2%. This study is consistent with [1, 15, 26].

The results show that keeping other things unchanged, the variable income of the household head has a positive and significant effect on food security status, indicating that household food security increases with higher level of a household monthly income. Regarding the marginal effect, keeping other factor unchanged, one birr increase in the income of the household head will result in an increase the probability of being food secure by about 55.7%. This study is in line with [27, 28].

## 5. Conclusion and Recommendation

### 5.1. Conclusion

Food insecurity is one of the major development challenges in Ethiopia. This study investigated the determinants of rural household food security status in the North Shewa region, Amhara region, Ethiopia. This study employed quantitative research approach and multistage sampling method was used to select 800 household heads from four districts, namely, Minjar Shenkora, Angolela Tera, Moretna Jiru, and Menz Gera districts. This study employed both descriptive and econometric methods of data analysis. In this study, food security status was calculated using the caloric intake method. The results show that, based on the food security threshold line 2200 kcal, a total of 482 (60.55%) sample households were food insecure in the study area. The logit estimated results reveal that age of the household head, education level of the household head, family size, off-farm activities, monthly income of the household, and distance from the market were identified as the major determinants of food security status in the study area.

### 5.2. Recommendation

Based on the findings, this study suggests the following recommendations; first, family size has a negative and significant effect on household food security status. Therefore, the government should sensitize households' heads on the practice of family planning to discourage larger household size. This can be achieved by integrated health and education services. Second, educational level of the household head has a positive and significant effect on household food security status. Policy measures are needed to encourage access education for households to enhance food security in the rural households. In response, regional, federal, and nongovernmental governments should make education accessible to rural households and providing training and other programs that can help families improve their food security.

Third, age of the household head has a positive effect on food security status of the rural household. This means younger households are less likely to be food secure. As a result, capacity building for young household heads should be given to the rural households.

Fourth, distance from the market has a negative and significant effect on food security. Hence, the government should expand and open new market area to farmers in rural areas. The local and regional governments can improve the role of market access for food security by constructing all-weather roads connecting kebeles to nearby markets. Finally, income of household and off farm activity participation has a positive and significant effect on rural household food security status. Therefore, the government and other stakeholders should support the rural household to increase their income alternatives and to improve food security through engaging in off-farm activities. In addition, food security strategies should be executed to improve households' monthly income earnings and to improve rural households' food security status.

## Figures and Tables

**Table 1:** Description, measurement, and expected sign of variables.

Variable	Variable value	Description	Expected sign
Dependent variable			
Household food security status	Dummy	(1 if “secure,” 0 if “insecure”)	
Independent variables			
Age	Continuous	Measured in years	Positive
Sex	Dummy	1 = male	Positive
Family size	Continuous	In number	Negative
Education level	Categorical	0 = No formal education, 1 = primary, 2 = secondary, 3 = above secondary school	Positive
Land size	Continuous	In hectare	Positive
Access to market	Continuous	Kilometer	Negative
Tropical livestock	Continuous	In number	Positive
Access to credit	Dummy	1 = if they had access	Positive
Off-farm activities	Dummy	1 = if they had participated	Positive
Saving	Dummy	1 = if they had saved	Positive
Income of the household	Continuous	In birr	Positive

**Table 2:** Summary statistics for continuous variables.

Variable	Mean	Std. dev.	Min	Max
Age	43.136	11.026	18	80
Family size	4.98	1.992	1	14
Cultivated land size	1.581	1.101	0.25	10
Distance from market	9.892	9.148	0.01	70
Tropical livestock	6.263	4.32	0	44.6
Household monthly income	3914.46	2266.349	321.6	14758.36

Source: own survey, 2021.

**Table 3:** Socioeconomic and institutional characteristics of the households.

Variable		Freq	Percent
Sex	Female	79	9.92
	Male	717	90.08
Access to credit	No	425	53.39
	Yes	371	46.61
Off-farm activity	No	493	61.93
	Yes	303	38.07
Saving	No	615	77.26
	Yes	181	22.74
Educational level	No formal education	309	38.82
	Primary school	385	48.37
	Secondary school	90	11.31
	Above secondary school	12	1.51

Source: own survey, 2021.

**Table 4:** Classifications of food security status.

food security status	Freq.	Percent
Food insecurity	482	60.55
Food secured	314	39.45
Total	796	100.00

**Table 5:** Factors affecting the probability of household-level food security status.

Logistic regression			Number of obs = 796	
			Lr chi2(10) = 829.37	
			Pseudo R-squared = 0.7767	
Log pseudo likelihood = −119.19659				
Variable	dy/dx	Std. Err.	z	Sig
Sex^*∗*^	0.010	0.017	0.560	

Age	0.002	0.001	2.120	^*∗∗*^

Education	0.021	0.010	2.100	^*∗∗*^

Family size	−0.133	0.034	−3.950	^*∗∗∗*^

Saving^*∗*^	0.023	0.020	1.140	

Off-farm activity participation^*∗*^	0.048	0.014	3.428	^*∗∗∗*^

Cultivated land	0.010	0.007	1.500	

Distance from market	−0.046	0.021	−2.190	^*∗∗*^

Credit access^*∗*^	0.005	0.013	0.420	

Tropical livestock	0.002	0.002	1.240	

Ln income	0.557	0.141	3.96	^*∗∗∗*^

^*∗∗∗*^ *p* < .01, ^*∗∗*^*p* < .05, ^*∗*^*p* < .1. (^*∗*^) dy/dx is for discrete change of dummy variable from 0 to 1.

## Data Availability

The facts that assisted the findings of this study are available upon reasonable request from the corresponding writer.
